# Clinical relevance of DNA microarray analyses using archival formalin-fixed paraffin-embedded breast cancer specimens

**DOI:** 10.1186/1471-2407-11-253

**Published:** 2011-06-16

**Authors:** Al Muktafi Sadi, Dong-Yu Wang, Bruce J Youngson, Naomi Miller, Scott Boerner, Susan J Done, Wey L Leong

**Affiliations:** 1 Ontario Cancer Institute and Campbell Family Institute for Breast Cancer Research, Princess Margaret Hospital, University Health Network, 610 University Avenue, Toronto, Ontario, M5G 2M9, Canada; 2 Laboratory Medicine Program, Princess Margaret Hospital, University Health Network, 610 University Avenue, Toronto, Ontario, M5G 2M9, Canada; 3 Department of Laboratory Medicine and Pathobiology, Faculty of Medicine, University of Toronto, 1 King's College Circle, Toronto, Ontario, M5S 1A8, Canada; 4 Department of Medical Biophysics, University of Toronto, Ontario Cancer Institute, Princess Margaret Hospital, 610 University Avenue, Toronto, Ontario, M5G 2M9, Canada; 5 Surgical Oncology, Princess Margaret Hospital, University Health Network, 610 University Avenue, Toronto, Ontario, M5G 2M9, Canada

## Abstract

**Background:**

The ability of gene profiling to predict treatment response and prognosis in breast cancers has been demonstrated in many studies using DNA microarray analyses on RNA from fresh frozen tumor specimens. In certain clinical and research situations, performing such analyses on archival formalin fixed paraffin-embedded (FFPE) surgical specimens would be advantageous as large libraries of such specimens with long-term follow-up data are widely available. However, FFPE tissue processing can cause fragmentation and chemical modifications of the RNA. A number of recent technical advances have been reported to overcome these issues. Our current study evaluates whether or not the technology is ready for clinical applications.

**Methods:**

A modified RNA extraction method and a recent DNA microarray technique, cDNA-mediated annealing, selection, extension and ligation (DASL, Illumina Inc) were evaluated. The gene profiles generated from FFPE specimens were compared to those obtained from paired fresh fine needle aspiration biopsies (FNAB) of 25 breast cancers of different clinical subtypes (based on ER and Her2/neu status). Selected RNA levels were validated using RT-qPCR, and two public databases were used to demonstrate the prognostic significance of the gene profiles generated from FFPE specimens.

**Results:**

Compared to FNAB, RNA isolated from FFPE samples was relatively more degraded, nonetheless, over 80% of the RNA samples were deemed suitable for subsequent DASL assay. Despite a higher noise level, a set of genes from FFPE specimens correlated very well with the gene profiles obtained from FNAB, and could differentiate breast cancer subtypes. Expression levels of these genes were validated using RT-qPCR. Finally, for the first time we correlated gene expression profiles from FFPE samples to survival using two independent microarray databases. Specifically, over-expression of *ANLN *and *KIF2C*, and under-expression of *MAPT *strongly correlated with poor outcomes in breast cancer patients.

**Conclusion:**

We demonstrated that FFPE specimens retained important prognostic information that could be identified using a recent gene profiling technology. Our study supports the use of FFPE specimens for the development and refinement of prognostic gene signatures for breast cancer. Clinical applications of such prognostic gene profiles await future large-scale validation studies.

## Background

Gene profiling is beginning to have an impact on personalized breast cancer care [1,2]. Gene expression profiling of breast cancers using DNA microarray technology is able to classify breast tumors into distinct biological subgroups and has been shown to predict treatment response and prognosis in several studies [3-9]. This high-throughput molecular technique requires fresh bio-specimens to allow extraction of RNA of sufficient quantity and quality for analysis. There are however limitations to the collection of fresh samples for prospective studies including time-sensitive tissue processing, lengthy patient accrual and follow-up; and bio-banks are not always readily available as a source of fresh frozen samples. Also, a requirement for fresh tissue to be used inevitably leads to a bias towards only larger tumors being studied. To overcome some of these critical shortcomings of prospective studies, the use of archival formalin-fixed paraffin-embedded (FFPE) samples offers a potential solution as most hospitals worldwide have collections of FFPE tumor specimens dating back many years. FFPE is the most widely used standard of practice for tissue fixation for the purpose of diagnostic histology and long-term storage.

The FFPE tissue preserving process was developed long before molecular biologists were concerned with the preservation of RNA. FFPE samples have not been considered a reliable source of RNA due to the tissue processing-associated degradation and chemical modifications of RNA. Formalin fixation creates cross-linking between nucleic acids and proteins and adds mono-methylol to amino groups on all four RNA bases [10]. Thus, a number of recent studies have started to look into the prospect of overcoming the RNA quality issues in FFPE specimens. Several studies used modification of standard RNA extraction methods to generate RNA of sufficient quality and quantity for DNA microarray analysis [11-14]. Some innovations in DNA microarray techniques were also reported [15,16]. A major breakthrough has been a new microarray technique developed by Illumina Inc. (San Diego, Ca), which involves cDNA-mediated annealing, selection, extension, and ligation (DASL), as well as random priming for detection of degraded RNA from FFPE samples [16-22]. This DNA microarray technique improved detection of fragmented RNA compared with conventional techniques. If the technique for gene profiling using FFPE specimens becomes sufficiently reliable, we anticipate that prognostic and predictive gene signatures can be identified using the vast available libraries of archival FFPE specimens with long-term treatment outcomes. In addition, any biomarker developed from FFPE samples could be more readily translated into clinical practice.

In this study, we aimed 1) to explore the feasibility of obtaining reliable microarray data from archival FFPE samples; 2) to compare gene expression profiles of FFPE samples with those of matched samples obtained from the same patients by FNAB; 3) to test the reproducibility of such experiments using quantitative real-time reverse transcription-polymerase chain reaction (RT-qPCR); and 4) to correlate the gene profiling of FFPE specimens with clinical outcome data using published microarray data sets.

## Methods

### Case selection and sample size

From the clinical tissue archive stored on site in the hospitals at University Health Network, a total of 50 FFPE blocks were evaluated by our breast pathologists (BY, NM, SD). Thirty nine blocks were finally selected for this study based on the following criteria: 1) sufficient invasive carcinoma remained in the FFPE block for RNA extraction and 2) > 70% malignant cells. In addition, 25 cases with FFPE blocks that also had available pre-operative fresh FNAB specimens that had been collected from an ongoing breast cancer gene profiling study in our institution on an unselected cohort of surgical patients. Clinically, the estrogen receptor (ER, also known as *ESR1*) and Her2/neu (Her2, also known as *ERBB2*) levels were evaluated by immunohistochemistry or by fluorescence in situ hybridization, according to the standard clinical protocols. Based on ER and Her2 status, the 25 cases were divided into four distinct subgroups: 10 cases in ER+/Her2-, 4 in ER+/Her2+, 6 in ER-/Her2+, and 5 in ER-/Her2- status for the next step of the analyses. The clinical descriptors for these 25 cases are provided in Table 1. The study protocol was approved by the Research Ethics Board at the University Health Network.

**Table 1:** The 25 patient and tumor characteristics.

Patient No.	Group No.	Patient Age	Tumor^1 ^Size	Tumor Grade	Positive^2 ^Nodes	ER	PR	Her2	FNAB RIN	FFPE RIN
1	E(+)H(-)-1	56	1.2	2	0(1)	+	+	-	7.5	1.7
2	E(+)H(-)-2	47	2.2	2	1(12)	+	+	-	9.2	2.3
3	E(+)H(-)-3	63	2.4	2	0(1)	+	+	-	7.0	2.5
4	E(+)H(-)-4	76	2.8	3	0(3)	+	+	-	8.9	2.5
5	E(+)H(-)-5	58	10.6	2	6(24)	+	+	-	6.6	2.5
6	E(+)H(-)-6	50	3.7	2	0(2)	+	+	-	8.6	2.4
7	E(+)H(-)-7	55	1.9	2	2(15)	+	+	-	9.4	1.5
8	E(+)H(-)-8	68	2.0	2	4(17)	+	+	-	7.2	2.7
9	E(+)H(-)-9	42	2.6	2	0(3)	+	+	-	7.8	2.3
10	E(+)H(-)-10	64	1.5	2	0(4)	+	+	-	7.4	2.1
11	E(+)H(+)-1	61	2.2	3	2(18)	+	-	+	8.3	2.4
12	E(+)H(+)-2	43	2.5	3	0(5)	+	-	+	7.9	2.4
13	E(+)H(+)-3	41	1.6	3	0(3)	+	+	+	9.3	2.2
14	E(+)H(+)-4	37	1.5	3	0(18)	+	+	+	9.0	2.1
15	E(-)H(+)-1	44	3.2	3	0(1)	-	-	+	6.7	2.7
16	E(-)H(+)-2	56	1.4	3	0(3)	-	-	+	6.9	1.9
17	E(-)H(+)-3	76	2.3	3	2(20)	-	-	+	5.6	2.3
18	E(-)H(+)-4	64	2.3	3	5(21)	-	-	+	8.8	2.2
19	E(-)H(+)-5	73	1.6	3	0(2)	-	-	+	6.5	2.4
20	E(-)H(+)-6	54	2.9	3	0(12)	-	-	+	8.0	2.5
21	E(-)H(-)-1	51	1.6	3	1(23)	-	-	-	8.3	1.7
22	E(-)H(-)-2	54	3.6	2	0(11)	-	-	-	6.9	2.4
23	E(-)H(-)-3	62	1.6	3	1(21)	-	-	-	6.6	2.4
24	E(-)H(-)-4	47	1.5	3	0(4)	-	-	-	5.6	2.3
25	E(-)H(-)-5	57	2.9	2	0(4)	-	-	-	8.9	2.2

E, ER; H, Her2; FNAB, fine needle aspiration biopsy; FFPE, formalin-fixed, paraffin-embedded tissue; RIN, RNA integrity number. ^1^Tumor size in cm, ^2^Total number of nodes removed shown in parenthesis.

### Tissue sampling and RNA extraction

FNAB samples and RNA extractions were prepared as previously described [23]. For FFPE samples, the selected blocks were sectioned at 10 μm thickness in an RNase-free environment. Total RNA was isolated from FFPE samples using a modified protocol described by Abramovitz et al [24] with RecoverAll Total Nucleic Acid kit (Ambion Austin, TX). In brief, the sections were deparaffinized with xylene and air dried tissue pellets were homogenized by overnight incubation at 50°C with Proteinase K in a lysis buffer. The Proteinase K was then inactivated by incubating the sample at 80°C for 15 minutes the next day. RNA was purified and extracted after a DNase I treatment using a spin column. Quantity of the RNA was measured by ND-1000 Spectrophotometer (Nanodrop Technologies, Wilmington, DE USA). To assess the quality and level of degradation of RNA, RIN (RNA integrity number) was assigned by the Agilent 2100 Bioanalyzer (Agilent Technologies, Santa Clara, CA, USA).

### Illumina whole genome direct hybridization and DASL assays

All Illumina related services were provided by The Centre for Applied Genomics at the Hospital for Sick Children (Toronto, ON). Illumina Human-Ref8 BeadChip V3 24 K whole genome gene chips (Illumina Inc., San Diego, CA, USA) were used for both direct hybridization and DASL assays. A direct hybridization assay was used for FNAB specimens by using Illumina's standard protocol http://www.illumina.com. DASL assays were used for FFPE specimens according to Illumina's standard protocol. In brief, the input amount of RNA for the DASL assays was 400 ng of total RNA obtained from FFPE blocks. The RNA was first converted to cDNA through a reverse transcription reaction with biotinylated primers. The biotinylated cDNA was then annealed to assay oligonucleotides, and bound to streptadivin conjugated paramagnetic particles. After the oligonucleotide hybridization, mishybridized and non-hybridized oligonucleotides were washed away. The hybridized oligonucleotides were then extended and ligated. These products formed a synthetic template that was transferred to a PCR reaction containing a fluorescently labelled primer. The labelled PCR product strand was then isolated, and the fluorescent products hybridized to a BeadChip. The BeadChip was then washed and imaged on the BeadArray Reader.

### Quantitative real-time reverse transcription-PCR (RT-qPCR)

The RNA expression level of the 14 most differentially expressed genes (Table 2) was assayed using RT-qPCR with TaqMan gene expression assays in a 7900 sequence detector (Applied Biosystems, Foster City, CA). One μg of total RNA from a FFPE specimen was reverse transcribed in a 20 μL final reaction volume, using SuperScript VILO cDNA synthesis kit. The amount of cDNA corresponding to 10 ng of RNA was used in 10 μL reactions with the TaqMan Universal PCR Master Mix and corresponding sequence-specific primers assay mix (Applied Biosystems). Human HeLa cell RNA was used as the calibration sample, and the housekeeping gene GAPDH served for the standardization of the individual PCR reactions.

**Table 2:** 14 selected differential expression genes.

Gene Symbol	The 25 breast cancers					NKI-295 dataset			GIS-251 dataset		
	**FNAB array**	**FFPE array**	**FFPE qPCR**	**FFPE vs. FNAB**	**FFPE vs. qPCR**	**Array**	**OS**	**DMFS**	**Array**	**OS**	**RFS**
	**P^1^**	**P^1^**	**P^1^**	**r^2^**	**r^3^**	**P^1^**	**P^4^**	**P^4^**	**P^1^**	**P^4^**	**P^4^**

AKR7A3	0.001	0.000	0.000	**0.701**	**0.653**	0.000	0.000	0.248	0.000	0.284	0.787
ANKRA2	0.001	0.009	0.021	**0.522**	0.390	0.000	0.021	0.071	0.000	0.064	0.139
ANLN	0.000	0.002	0.007	**0.731**	**0.543**	0.000	0.002	0.010	0.000	0.009	0.004
CA12	0.000	0.000	0.000	**0.607**	**0.710**	0.000	0.010	0.299	0.000	0.358	0.468
DNALI1	0.000	0.000	0.000	**0.681**	**0.646**	0.000	0.009	0.097	0.000	0.565	0.582
ERBB2	0.000	0.008	0.000	0.370	**0.815**	0.000	0.005	0.024	0.000	0.006	0.001
ESR1	0.000	0.000	0.000	**0.787**	**0.862**	0.000	0.004	0.030	0.000	0.698	0.742
FUT3	0.001	0.001	0.003	**0.542**	0.444	0.000	0.061	0.009	0.000	0.023	0.287
GREB1	0.001	0.001	0.000	**0.732**	**0.711**	0.000	0.054	0.667	0.000	0.023	0.487
KIAA1407	0.000	0.000	0.010	**0.460**	0.337	0.000	< 0.001	0.000	0.000	0.006	0.197
KIF2C	0.000	0.001	0.020	**0.710**	**0.647**	0.000	< 0.001	0.000	0.000	0.022	0.004
LONRF2	0.001	0.007	0.004	**0.398**	**0.588**	0.000	0.001	0.168	0.000	0.294	0.393
MAPT	0.000	0.000	0.000	**0.740**	**0.677**	0.000	0.007	0.023	0.000	0.001	0.014
PGR	0.000	0.000	0.000	**0.870**	**0.674**	0.000	< 0.001	< 0.001	0.000	0.022	0.206

FNAB, fine needle aspiration biopsy; FFPE, formalin-fixed, paraffin-embedded tissue; qPCR, Quantitative RT-PCR; OS, Overall survival; DMFS, Distant metastasis free survival; RFS, Relapse free survival; ^1^*P*-value of ANOVA test among the 4 ER/Her2 groups; ^2^Correlation coefficient, Pearson correlation test between FFPE array and FNA array (value in boldface type is statistically significant, *P *< 0.05); ^3^Correlation coefficient, Pearson correlation test between FFPE array and FFPE qPCR (value in boldface type is statistically significant, P < 0.05); ^4^*P-*value of Log-rank Test.

### Data analysis

Scanned microarray image data were used to process expression data by Illumina Gene Expression Module of GenomeStudio Software. The microarray gene expression data was normalized using background subtraction and Quantile methods. Sequence Detection Software (Applied Biosystems) was used to obtain the RT-qPCR amplification plots to quantify gene expression values using the cycle threshold method. All data were represented as log_2 _ratios for the expression analysis of gene transcriptions. ANOVA and t-test were used to evaluate the variant significance of gene expression in different groups. Pearson correlation was used to measure the expression similarities between FNAB and FFPE specimens as well as between the microarray and RT-qPCR levels. Hierarchical clustering analysis was used to present gene expression patterns. Kaplan-Meier analysis was used to compare patients' survivals in differential gene expression groups, and the differences were determined by the log-rank test. The microarray data have been deposited in NCBI's Gene Expression Omnibus http://www.ncbi.nlm.nih.gov/geo and are accessible through GEO series accession number GSE23386. Two publicly available microarray datasets from 295 breast cancers of the Netherlands Cancer Institute (NKI-295) [7], and from 251 breast cancers of the Genome Institute of Singapore (GIS-251) [25] were used for validation analyses.

## Results

### Quality and quantity of FFPE RNA

**Figure 1:**
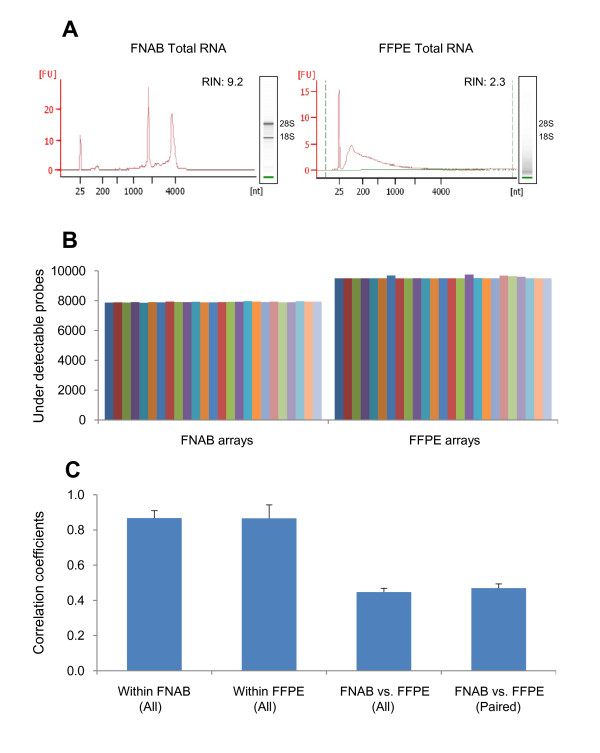
**Comparison of total RNA and microarray signals between FNAB and FFPE specimens**. (A) Comparison of Agilent 2100 Bioanalyzer analysis of total RNA between FNAB and FFPE from a breast cancer sample E(+)H(-)-2. (B) Noise levels of the microarray signals. The average numbers of under-detectable probes from Illumina Human-Ref8 24 K BeadChip are 7906.8 ± 27.9 in 25 FNAB arrays and 9531.4 ± 74.3 in 25 FFPE arrays, respectively. (C) Reproducibility of microarray signal. The averages of Correlation Coefficients are 0.87 ± 0.04 within 25 FNAB arrays, 0.87 ± 0.08 within 25 FFPE arrays, 0.45 ± 0.02 between the 25 FNAB arrays and 25 FFPE arrays, and 0.47 ± 0.02 between the 25 paired FNAB and FFPE arrays, respectively.

Initially, as a pilot study, we extracted RNA from FFPE specimens of eight human mammary reduction mammoplasty cases and generated DNA microarray data by using the DASL assay and Illumina Genome Wide HumanRef-8 BeadChip. When we compared the gene expression signals between the replicates, the average correlation coefficient was as high as 0.96 ± 0.02 (data not shown). In this study, we evaluated 50 FFPE blocks from the clinical surgical pathology, and total RNA was extracted from 39 FFPE blocks. Eleven cases were excluded due to small tumor size or unavailability of a suitable FFPE block. Average total RNA yielded from FFPE samples was 4.3 μg (range 0.5 - 10 μg). Spectophotometric 260/280 ratio of extracted RNA samples ranged from 1.5 - 2. As expected, the landmark ribosomal peaks (18S and 28S) were not well detected in the FFPE samples using Agilent 2100 Bioanalyzer (Figure 1A). However, samples from 32 cases (82%) had RIN values above 1.5 and were considered suitable for subsequent DNA microarray analyses [17].

### Comparison of RNA and array signal between FFPE and FNAB

Out of the 32 FFPE samples, 25 had corresponding FNAB specimens. We performed DASL assay and subsequent DNA microarray hybridization on the 25 paired FFPE samples. The 25 paired FNAB RNAs were used for direct hybridization assays to the same Illumina HumanRef-8 V3 platform. As expected, RNA from FFPE samples had lower RIN values than that of RNA extracted from fresh FNAB (Table 1 and Figure 1A). In all 25 breast cancers, when we compared between the two different specimen types, gene expression status from FFPE specimens tended to have higher noise levels defined by a higher number of gene probes not detectable during the DNA microarray analyses (Figure 1B). When compared within the two specimen types, the expression signals within both FFPE samples and FNAB had similarly high average correlation coefficients (0.87 ± 0.04 and 0.86 ± 0.08, respectively) supporting the reproducibility of the DNA microarray data generated within each specimen type. They were less similar between specimen types with the average correlation coefficients of 0.45, which was likely due to the dominant effects on the RNA related to the tissue processing for FFPE specimens and/or the different hybridization techniques during DNA microarray analyses (Figure 1C).

### Comparison of ER and Her2 expression between FFPE samples and FNAB

**Figure 2:**
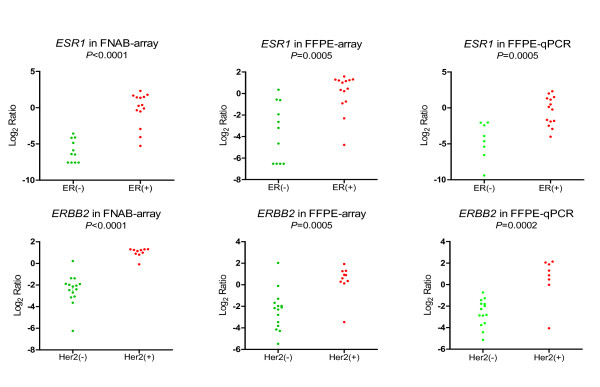
**Comparison of ER and Her2 clinical status to array and RT-qPCR**. The clinical status of ER and Her2 reported by immunohistochemistry or fluorescence in situ hybridization were compared with *ESR1 *and *ERBB2 *levels from expression microarrays. From top to bottom: FNAB specimens (FNAB-arrays), FFPE samples (FFPE-arrays) of the 25 paired breast cancers, and RT-qPCR expression in FFPE samples (FFPE-qPCR) of the 22 available breast cancers in scatter dots plots. The *P *values were calculated by *t*-Test.

To determine whether FFPE samples could yield the same biologically and clinically relevant information as the FNAB, ER and Her2 clinical status detected by immunohistochemistry or fluorescence in situ hybridization were compared to the expression levels of *ESR1 *and *ERBB2 *in microarray expression and RT-qPCR analyses (Figure 2). The two clinical receptors' status remained reproducible as illustrated by a very strong correlation within the two specimen types FFPE (*P *= 0.0005) and FNAB (*P *< 0.0001) in the microarray expression level. As well, the two clinical receptors were also validated by using RT-qPCR in FFPE specimens (*P *= 0.0002).

### Comparison of gene profiling between FFPE samples and FNAB

**Figure 3:**
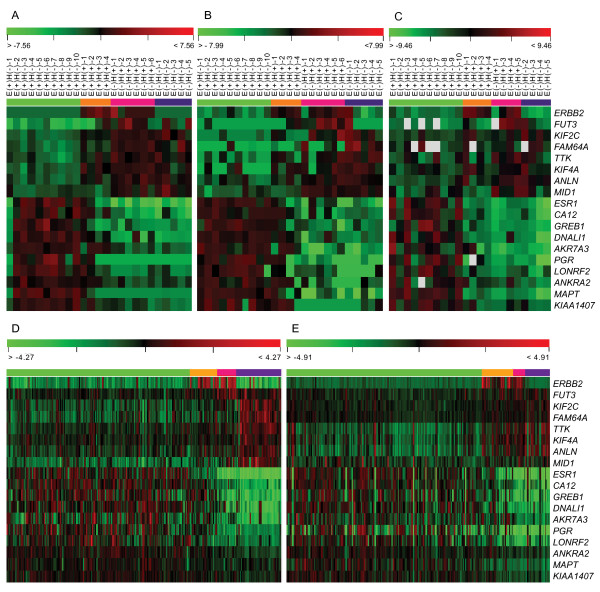
**Comparison of the gene expression profiles in different datasets**. The expression profiles of the 18 comparable differentially expressed genes were compared in FNAB array (A) and in FFPE array (B) of the 25 breast cancers, and in FFPE RT-qPCR of the 22 breast cancers (C), as well as in the validation NKI datasets of 295 breast cancers (D) and validation GIS dataset of 251 breast cancers (E). Among the four subtype of breast cancers, samples were indicated as E(+)H(-) (ER+/Her2-, green), E(+)H(+) (ER+/Her2+, orange), E(-)H(+) (ER-/Her2+, pink) and E(-)H(-) (ER-/Her2-, blue), respectively.

To further demonstrate whether gene profiles generated by FFPE and FNAB arrays can differentiate tumors into their different ER/Her2 subtypes, we performed an ANOVA test to determine the genes that were most differentially expressed among the four distinct subtypes. As a result, 485 differentially expressed probes from FNAB arrays (*P *< 0.001) and 258 probes from FFPE arrays (*P *< 0.01) were obtained, of which 39 probes (representing 38 genes) overlapped between the two specimen types. The fact that all these 38 overlapped genes were differentially expressed in the same manner in both specimen types allowed us to directly compare the expression patterns between the two. A hierarchical clustering analysis using the 39 overlapped probes (data not shown) and a subset of 18 genes for both array and RT-qPCR analyses (see the section below for details) showed an identical expression pattern differentiating the four subtypes using FNAB arrays (Figure 3A) and FFPE arrays (Figure 3B).

### Validation of gene profiling

Since most of the publicly available microarray data were generated using fresh tumor specimens, we tested the 38 genes that were reproducible between fresh and FFPE specimens to see if their expression pattern could be correlated with long-term clinical outcome data available in these public databases. Out of the 38 genes, 28 genes were reported in the two public databases NKI-295 [7] and GIS-251 [25]. Interestingly, these genes were differentially expressed in a similar fashion when we divided the tumors into the same four tumor subtypes (ANOVA test, *P *< 0.001). In addition, out of 28 genes, 21 were significantly correlated to patients' survivals (Log-rank test, *P *< 0.05). Consequently, the 21 genes were chosen for further RT-qPCR analysis for validation of the array expression on FFPE specimens. Unfortunately, by using the commercial RT-qPCR reactions (TagMan, Applied Biosystems), three RT-qPCR reactions out of the 21 genes did not produce any readable signals, and three samples out of 25 tumors failed to amplify in most of the RT-qPCR reactions. As the result, the remaining 18 genes and 22 FFPE specimens that were successful in RT-qPCR were used for subsequent analyses. When comparing the differential expression of the 18 genes, we found a near-identical gene expression pattern in FNAB arrays (Figure 3A), FFPE arrays (Figure 3B) and FFPE RT-qPCR (Figure 3C), as well as NKI-295 arrays (Figure 3D) and GIS-251 dataset (Figure 3E). Out of the 18 genes detected by RT-qPCR in the 22 FFPE specimens, 14 genes were significantly differentially expressed among the four clinical subtypes (ANOVA test, *P *< 0.05) as well as their array expression by using both FNAB and FFPE specimens, and in the two validation datasets. The final 14 genes are listed in Table 2.

### The biomarkers for prognosis of breast cancers

**Figure 4:**
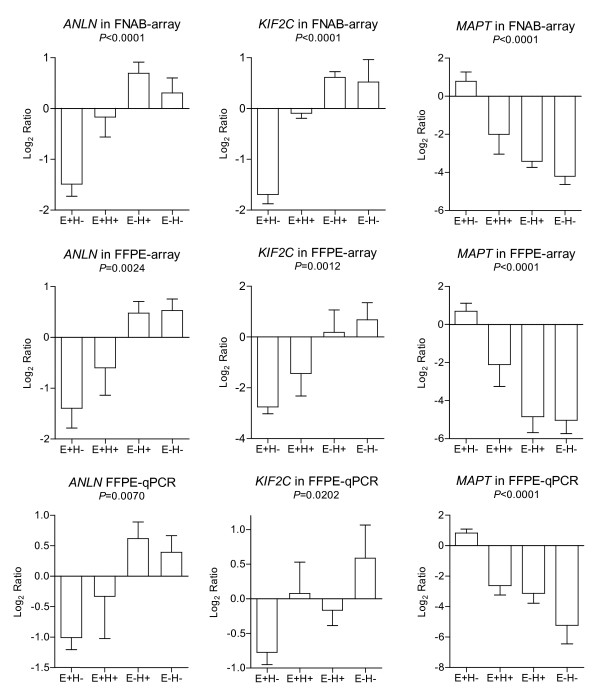
**Differential expression of the biomarkers**. The histograms show the comparison of the average expression values of the three prognostic genes *ANLN, KIF2C *and *MAPT *from left to right respectively, which were shown to be significantly different among the four ER/Her2 clinical status groups [ER+/Her2- (E+H-), ER+/Her2+ (E+H+), ER-/Her2+ (E-H+), and ER-/Her2- (E-H-)], but reproducible among the microarray data from FNAB (FNAB-array) and FFPE samples (FFPE-array) of the 25 paired breast cancers, and RT-qPCR expression in FFPE samples (FFPE-qPCR) of the 22 available breast cancers. The *P *values were calculated by ANOVA test.

**Figure 5:**
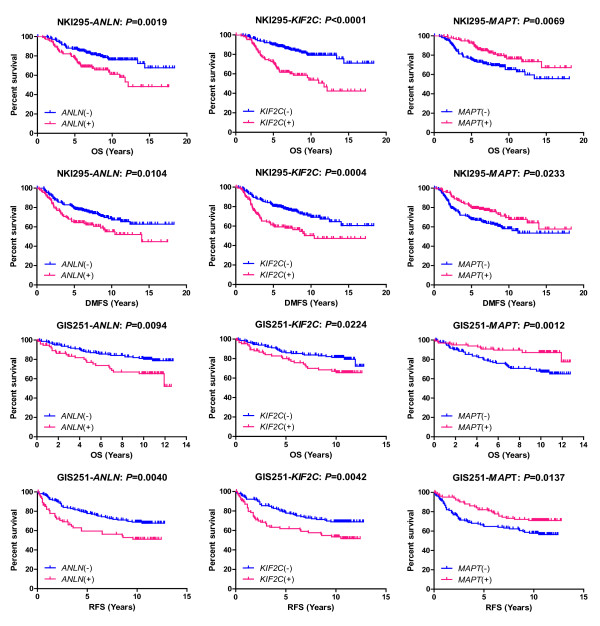
**Kaplan-Meier survival curves**. The association between the gene expression of *ANLN*, *KIF2C *and *MAPT*, from left to right respectively, and the clinical outcomes in patients' overall survival (OS) or distant metastasis free survival (DMFS) from the 295 patients in the NKI validation dataset (NKI295), and OS or relapse free survival (RFS) from the 251 patients in the GIS validation dataset (GIS251), from top to bottom respectively. *P *values were calculated by log-rank test.

When we divided the tumors into the four clinical subtypes, all 14 genes were differentially expressed in a similar fashion in all three tests analyses (FNAB, FFPE and RT-qPCR) in the training sets and the two independent published microarray datasets, served as validation sets. When looking at the survival data from the two validation datasets, these 14 genes showed a significant association between their expression levels in the breast cancers and the clinical outcome of the patients: overall survival and distant metastasis free survival in 295 patients from the NKI dataset, and overall survival and relapse free survival in 251 patients from the GIS dataset (Table 2). These genes could be used as potential biomarkers for predicting the clinical outcomes in breast cancer patients, in both fresh FNAB specimens and archival FFPE specimens. Specifically, two genes, *ANLN *(anillin, actin binding protein) and *KIF2C *(kinesin family member 2C) were under-expressed in ER+ tumors and over-expressed in Her2+ and ER-/Her2- tumors; whereas *MAPT *(microtubule-associated protein tau) was over-expressed in ER+ tumors and under-expressed in Her2+ or ER-/Her2- tumors (Figure 4). The over-expression of *ANLN *and *KIF2C*, and the under-expression of *MAPT *consistently showed a strong correlation with poor survival in the breast cancer patients from both validation datasets (Figure 5). These findings demonstrated that we can generate informative microarray data from FFPE specimens, and the expression levels of a subset of genes are reproducible and informative when compared to FNAB specimens. The prognostic information of these genes is preserved in FFPE specimens.

## Discussion

Since Rupp and Locker [26] reported their first successful RNA extraction from FFPE specimens in 1988, significant strides have been made to enable RNA profiling from FFPE tissues, including efforts to standardize tissue handling and fixation procedures and improving RNA extraction methodologies [27,28]. Very few technologies have emerged despite these advancements that are capable of whole transcriptome profiling from archived FFPE material [24,29,30]. Initial attempts at DNA microarray analyses using FFPE samples yielded poor reproducibility [31], or loss of detection of gene signatures when compared with matched fresh samples [32]. Several commercial and academic endeavours have recently become more successful in their ability to retrieve meaningful biological information from degraded FFPE-derived RNAs [15,21,22,24,33-36].

The DASL assay incorporates random priming during cDNA synthesis, and therefore does not depend solely on the polyA/oligo-dT based priming process used in conventional DNA microarray methods. In addition, the assay requires a relatively short target sequence of about 50 nucleotides to query oligonucleotide annealing; which improved its ability to quantify fragmented RNA species [37]. Using these technologies, we evaluated the feasibility of using FFPE samples for DNA microarray analyses. Initially, as a pilot study, RNA from FFPE blocks of human mammary reduction mammoplasty tissues were extracted and used to generate DNA microarray data using DASL assay and Illumina HumanRef8 BeadChip, a genome-wide gene panel that contained over 24 thousands genes. Within eight replicates of the same type of specimens, the average correlation coefficient of array expression signals was very high at 0.96 ± 0.02 which showed an impressive technological reproducibility. This is similar to a recent study by Ton et al who reported a high correlation (r = 0.98) among 12 technical replicates by using the DASL technology [21].

We carried out our current study using human breast cancer specimens to test the feasibility of performing such experiments with a future plan to extend the technology to a large clinical library of breast cancers. Using RNA extraction methods described recently [23], we found that over 80% of the RNA extracted from archival FFPE tumor samples could be used for subsequent DASL analyses and produced DNA microarray data that were informative and could be validated and compared to that obtained from fresh FNAB specimens collected from the same patients.

In this study, we demonstrated a high reproducibility of the DNA microarray data when comparing the array signals within the FNAB or FFPE tumor specimens (r = 0.87). The comparison between the two specimen types yielded lower correlation coefficients (r = 0.45), which was likely due to dominant effects on the RNA related to the tissue processing for FFPE specimens and the different hybridization techniques in DNA microarray protocols (Figure 1C). Nevertheless, some clinically relevant microarray data remained reproducible as illustrated by a very strong correlation between the microarray expression levels of *ESR1 *and *ERBB2 *to the clinical status of ER and Her2 respectively in both FFPE and FNAB samples, which were also validated with RT-qPCR in FFPE specimens. This result was consistent with two recent studies by Ton et al [21] and Mulligan et al [22]. The gene profiling of FFPE samples suffered from a higher ratio of noise-to-signal (Figure 1B) and thus detected a smaller number of differentially expressed genes compared to FNAB samples (258 vs. 485). Therefore, we anticipate that the identification of subtle changes in expression levels in FFPE samples will remain challenging using current technologies.

To date, high-throughput gene expression profiling has demonstrated the potential uses of gene profiling as molecular subtype classifiers [6], prognostic indicators [7,8], and treatment predictors [9] by using fresh breast cancer specimens. If gene profiling becomes a standard part of the pathological assessment of a tumor in the future, the use of FFPE material would be advantageous, as it obviates the need for fresh bio-specimens that can be very time sensitive and often impractical to collect in routine clinical settings. In certain clinical situations, including recurrent diseases and evaluation of long-term endocrine therapy, when there is no fresh bio-specimen from the original primary tumors; gene profiling using FFPE may provide an alternative to allow evaluation of the prognostic or predictive gene profiles.

The use of FFPE samples for gene profiling studies has been considered suboptimal in the past due to concerns about RNA degradation and less than 5% of all microarray studies to date have been reported using archival tumor tissues that were formalin-fixed and paraffin-embedded. Very few microarray studies have been conducted to investigate the gene profiling of FFPE samples and compared the results with their matched fresh frozen tissue. Some data is available in carcinomas of colon, liver and breast [4,5,15,35]. In breast cancer, Da Silva et al [20] used FFPE samples from invasive lobular carcinoma only to check the expression profile of E-cadherin. Ravo et al. [17] used RNA from cultured cell lines, cryopreserved tumors and FFPE samples from breast cancers. Waddell et al. [19] and Bibikova et al. [16] included both fresh frozen and FFPE samples in their studies to compare the gene profiling of breast cancers in both type of tissues. However, their studies focused only on the technical aspect without any clinical correlations. We have correlated our gene profiling results to clinical receptor status, and demonstrated the ability to use gene expression profiling from FFPE to differentiate tumors from 4 distinct tumor subtypes. We identified a set of 38 genes that were reproducibly able to identify the different clinical groups in both FFPE and FNAB specimens. These findings suggested that there are subsets of genes in FFPE specimens that could maintain their clinical relevance despite the tissue processing effects. Interestingly, subsets of these genes were correlated to clinical outcomes as demonstrated in our study using publicly available microarray validation databases.

Although we are the first to show that the over-expression of *ANLN *and *KIF2C*, and the under-expression of *MAPT *predict for poor survival in patients with breast cancer (Figures 4 and 5), there is some evidence that supports the correlation of these three genes with prognosis and carcinogenesis in other cancers, and treatment in breast cancers. The over-expression of *ANLN *has been reported to be a biomarker for pancreatic carcinoma [38], and predicted for poor survival in early lung cancers [39]. Shimo et al reported that the over-expression of *KIF2C *might be involved in breast carcinogenesis and is a therapeutic target for breast cancers [40]. The expression of *MATP *has been correlated to the sensitivity to chemotherapies in breast cancer [41,42]. These results strongly support that *ANLN*, *KIF2C*, and *MAPT *could play a role in the carcinogenesis, treatment and prognosis of breast cancers.

FFPE samples are widely available and can be linked to clinical outcome information, often available through institutional or provincial cancer registries. The availability of large libraries of archival FFPE tissue samples could potentially solve some of the most critical challenges that investigators face when using prospectively collected specimens, namely, patient recruitment and expensive long-term follow-up. Validation studies can also be done fairly quickly by selecting an independent patient population annotated with critical long-term clinical outcome data. Once the technology for gene profiling using FFPE matures, it will likely play an important role in the clinical management of breast cancer patients.

## Conclusion

There is tremendous potential in using FFPE specimens for gene profiling, especially in breast cancer, in which there are existing data to support the prognostic and predictive implications of tumor gene profiles. Based on the comparison of gene profiling on FFPE breast cancer specimens and matched fresh specimens, we can conclude that important clinically relevant information can be identified using FFPE specimens and we even demonstrated the potential for using the microarray data to be used as a prognostic tool. Further improvements in current DNA microarray technologies will likely to bring gene profiling of breast cancer into routine clinical practice as we move towards personalized breast cancer care.

## Competing interests

The authors declare that they have no competing interests.

## Authors' contributions

The study was designed by WLL and SJD and conducted by AMS. DYW performed data analyses, and manuscript submission. Manuscript was prepared by AMS, edited and modified by WLL, DYW and SJD, and all authors proofread the manuscript. SJD, BJY, NM and SB performed the pathology for the study.

## Pre-publication history

The pre-publication history for this paper can be accessed here:

http://www.biomedcentral.com/1471-2407/11/253/prepub
